# Differentially expressed microRNA cohorts in seed development may contribute to poor grain filling of inferior spikelets in rice

**DOI:** 10.1186/s12870-014-0196-4

**Published:** 2014-07-23

**Authors:** Ting Peng, Hongzheng Sun, Mengmeng Qiao, Yafan Zhao, Yanxiu Du, Jing Zhang, Junzhou Li, Guiliang Tang, Quanzhi Zhao

**Affiliations:** 1 Collaborative Innovation Center of Henan Grain Crops, Henan Agricultural University, Zhengzhou 450002, China; 2 Research Center for Rice Engineering in Henan Province, Henan Agricultural University, Zhengzhou 450002, China; 3 Department of Biological Sciences, Michigan Technological University, Houghton 49931, Michigan, USA

**Keywords:** Rice (Oryza sativa), microRNA, Differential expression, miRNA dynamics, Inferior spikelets, Superior spikelets, Grain filling

## Abstract

**Background:**

The inferior spikelets are defined to be those at portions where the grains receive less photosynthetic products during the seed development. The typical inferior spikelets are physically located on the proximal secondary branches in a rice panicle and traditionally characterized by a later flowering time and a slower grain-filling rate, compared to those so-called superior spikelets. Grains produced on the inferior spikelets are consequently under-developed and lighter in weight than those formed on the superior spikelets. MicroRNAs (miRNAs) are recognized as key players in regulating plant development through post-transcriptional gene regulations. We previously presented the evidence that miRNAs may influence grain-filling rate and played a role in determining the grain weight and yield in rice.

**Results:**

In this study, further analyses of the expressed small RNAs in superior and inferior spikelets were conducted at five distinct developmental stages of grain development. Totally, 457 known miRNAs and 13 novel miRNAs were analyzed, showing a differential expression of 141 known miRNAs between superior and inferior spikelets with higher expression levels of most miRNAs associated with the superior than the inferior spikelets during the early stage of grain filling. Genes targeted by those differentially expressed miRNAs (i.e. miR156, miR164, miR167, miR397, miR1861, and miR1867) were recognized to play roles in multiple developmental and signaling pathways related to plant hormone homeostasis and starch accumulation.

**Conclusions:**

Our data established a complicated link between miRNA dynamics and the traditional role of hormones in grain filling and development, providing new insights into the widely accepted concepts of the so-called superior and inferior spikelets in rice production.

## Background

Rice (*Oryza sativa L.*) is one of the most important food crops in the world, providing calories for over 21% of global population and 76% of South East Asian [1]. The yield of rice is determined primarily by two vital factors, the grain filling rate and subsequently the grain weight [2]. It has been demonstrated by many researches that grain weight and the grain plumpness (a parameter describing the grain quality) are mostly positional-dependent in a rice panicle [3]–[5]. A rice panicle is composed of numerous branches termed spikelets with some having high quality seeds termed superior spikelets and some with poor quality seeds termed inferior spikelets. In general, the superior spikelets flower earlier and subsequently have a faster grain-filling rate in seed development, producing high-quality seeds. The flowers or seeds on superior spikelets are typically located on apical primary branches in a panicle. In contrast, the inferior spikelets flower later with a lower grain-filling rate and are located on the proximal secondary branches, resulting in low-quality seeds [3],[6],[7].

MicroRNAs (miRNAs), a type of endogenous non-coding small RNAs produced from stem-loop structured precursors, have been proved to play curial roles in many aspects of plant development, such as organ morphogenesis [8],[9], stress response [10],[11], flowering control [12],[13], phytohormone homeostasis [14],[15], and grain/fruit development [16],[17]. The expressions of various miRNAs are extremely dynamic during rice grain development, revealed by a series of studies utilizing small RNA high-throughput sequencing technology [16],[18]–[21]. For example, in one study, most miRNAs were shown to be equally expressed or expressed much higher in grains of 6–10 days than those of 1–5 days after fertilization [20]. A similar study showed that a high proportion of the detected miRNAs were up-regulated in seeds of 5 to 7 days after fertilization [19]. The analysis of 445 known miRNAs and 45 novel miRNAs in our previous studies suggests the expressions of these miRNAs in rice grain were in a developmental stage dependent manner. The expressions of most known miRNAs increased gradually as rice grain filling went on [16]. These observations were also in contrast to another study showing that about half of the known miRNAs were up-regulated, while the remaining miRNAs were down-regulated during indica rice grain development [18].

Although it is not known yet if all the known miRNAs are involved in grain development, certain specific miRNAs showed a high correlation with the grain developmental process. MiR167 is such a candidate miRNA that may plays a role in rice grain filling through the auxin-miR167-*ARF8*-*OsGH3.2* regulatory pathway [21]. Over-expressing miR167 significantly reduced plant height, tiller number of individual plant, panicle length, spikelet number of each panicle, and seed setting rate via regulating its target ARF family transcriptional factors [22]. MiR397, miR398, miR408 and miR528 are potential grain filling regulators via controlling the levels of their target genes which encode copper-binding proteins and/or L-ascorbate oxidases [21]. MiR397 is highly expressed in rice young panicles and grains, and over-expressed miR397 enlarged the grain size, promoted panicle branching, and significantly increased rice grain yield via down-regulating its target *OsLAC*[17]. *OsSPL14*, a target of miR156, contributes to generating ideal rice plant architecture with a reduced tiller number, increased lodging resistance and enhanced grain yield [23],[24]. *OsSPL16*, another target of miR156, plays crucial roles in regulating grain size, shape and quality as well [25]. Furthermore, the seed size was reduced in miR393-overexpressed transgenic lines when compared with that of the wild-type [26]. All these results indicate that miRNAs play important roles in rice grain development or rice grain filling.

We have previously found 351 and 312 known miRNAs expressed in superior and inferior grains respectively and specifically at 18 days after flowering (DAF). Among them, 189 miRNAs were found differentially expressed between grains from superior and inferior spikelets, suggesting their potential roles in multiple physiological or metabolic processes during rice grain development [4]. To further investigate their dynamic roles of miRNAs in determining the development of superior and inferior spikelets during all stages of the entire rice grain filling process, we sequenced and analyzed miRNAs in superior and inferior spikelets of five stages at 10, 15, 21, 27, 35DAF, respectively. As a result, 457 known miRNAs, 13 novel miRNAs were revealed. Those known miRNAs that differentially expressed between superior and inferior spikelets were specifically studied and analyzed in clustering. We found that the target genes of some key miRNAs constituted essential regulatory networks in controlling various metabolic processes, including hormone homeostasis and starch accumulations. Most importantly, we found that the occurrence of inferior spikelets was tightly associated with the lower expressions of miRNAs that were differentially expressed between superior and inferior spikelets. Our results suggest a vital role of miRNA networks and their expression levels in determining the rice grain filling and provide mechanisms for the formation of grain weight in superior and inferior spikelets.

## Results

### Physiological and phenotypic differences between superior and inferior spikelets

**Figure 1:**
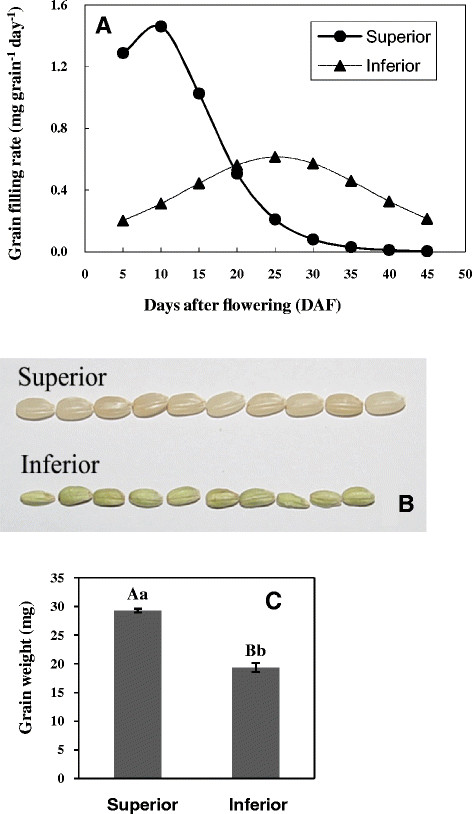
**The differences between superior and inferior spikelets by grain filling rate, grain appearance, and the grain weight in rice. (A)**. A line chart showing the grain filling rate difference between superior and inferior spikelets at different days after flowering (DAF) during rice grain filling. Grain filling rate was calculated by regression analysis using Logistic equation from the average of five repeats of the grain weight as described in Method. **(B)**. Distinct appearance and comparison of grains in superior and inferior spikelets of brown rice. Grain size and plumpness can be visually compared in this figure. **(C)**. Bar graph showing the statistical comparison of the final grain weight between superior and inferior spikelets. Equal amount of grains from triplicate lines were used for this measurement and error bar represented the standard errors of the means. Student two-tailed t test was used for statistical analysis of the difference; Aa and Bb indicate that the difference is of dramatic significance.

In general, the rice grain filling processes were dynamic in spikelets according to their locations on the rachis/panicle branches. Superior spikelets are on the top of the panicle, always flower earlier, have grains filling faster, and the higher final grain weight and plumpness than the inferior spikelets which are located on the base of the panicle [3],[6],[7]. Specifically, grain filling rates of superior spikelets (1.3-1.4 mg per grain per day) at the initial 5 to 10 DAF are 4 times faster than those of the inferior spikelets (0.2-0.3 mg per grain per day). Within 20 DAF, the majority of the grains in superior spikelets were filled up at the highest rate and then the filling rate dropped to a level that is comparable to the highest filling rate of the inferior grains (Figure 1A). In comparison, the grain filling rate in inferior spikelets (~0.6 mg per grain per day) was only less than 50% of the highest filling rate of the superior spikelets (~1.5 mg per grain per day) at this time point. The overall process of grain filling of superior spikelets took about 30 days with a much higher filling rate showing an asymmetrical curve, whereas that of the inferior spikelets took 45 days with a significantly lower filling rate showing a symmetrical normal curve (Figure 1A). Consequently, grains from superior spikelets were phenotypically larger and fully plump, while those from inferior spikelets appeared smaller with partial filling (Figure 1B and C).

### Differential expressions of overall small RNAs between superior and inferior spikelets

There are two major populations of small RNAs that have been identified in plants according to their lengths: 21-nucleotide (nt) and 24-nt small RNAs. In order to understand how these two types of small RNAs are expressed in superior and inferior spikelets during rice grain filling, high throughput RNA sequencing technology was employed to the small RNA libraries made from both types of spikelets. Ten samples of superior and inferior grains at 10, 15, 21, 27, and 35 DAF were used to isolate small RNAs for sequencing. After trimming adaptor sequences and removing those reads with low quality and lengths smaller than 18 nucleotides, about 10,658,388 to 17,702,636 high-quality small RNA reads, representing the lowest 3,518,252 to the highest 4,917,105 distinct small RNAs, were obtained from each library. Among those, more than 2,826,852 distinct reads (77.18%) were perfectly matched to the rice genome by analysis using SOAP (Additional file 1) [27].

**Figure 2:**
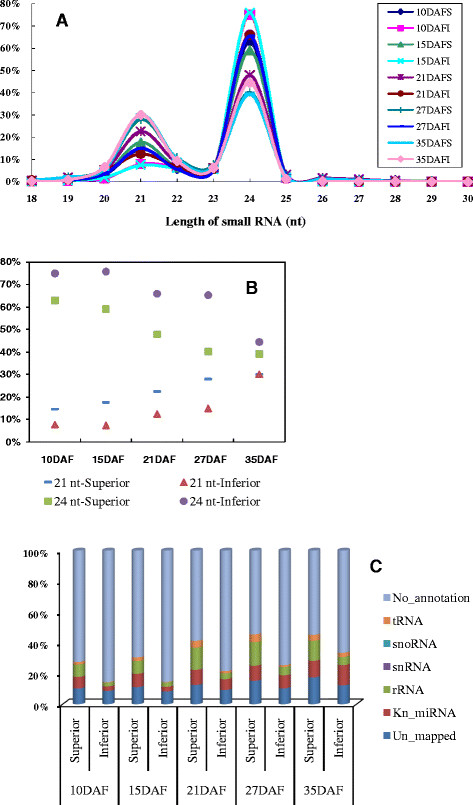
**Overall small RNA dynamics during rice grain filling processes. (A)**. Line charts showing the length-distribution of total small RNAs from 10 deep sequenced libraries of both superior and inferior spikelets at different rice grain filling stages. **(B)**. Dot chart showing different percentages of the 21- and 24-nt total small RNAs presenting in superior and inferior spikelets at different rice grain filling stages. **(C)**. Bar graph showing the small RNA dynamics of different categories from the 10 libraries of different grain filling stages.

Among millions of high-quality small RNAs from the individual libraries, the 24-nt and 21-nt small RNAs were dominant in all cases (Figure 2A and B). Specifically, 58.28% and 18.30% of the total reads were 24-nt and 21-nt small RNAs in the developing seeds from the ten libraries. In contrast to the report that 21-nt small RNAs were the most abundant population present in leaf and tricellular pollen in rice [28], our data showed that 24-nt small RNAs occupied the highest percentage in both superior and inferior spikelets (Figure 2A and B), which may be due to tissue- and temporal- specific expressions of small RNAs during rice organ development. Furthermore, there are more 24-nt small RNAs present in inferior spikelets than in the superior ones at all grain-filling stages, whereas the 21-nt small RNAs showed a reversed trend (Figure 2B).

Among the above-mentioned small RNAs, miRNAs as well as other types of small RNAs were analyzed. For example, all the known miRNAs were identified by comparing the total small RNAs with the known miRNAs stored in miRBase (release 17.0), and their abundances were analyzed. We observed that a higher percentage of total known miRNA was expressed in superior spikelets than in inferior spikelets during most rice grain filling stages except for the one at 35 DAF, at which no obvious difference was found between superior and inferior spikelets (Figure 2C, Additional file 1). Other types of small RNAs that were characterized for each dataset were mainly associated with rRNAs, snRNAs, snoRNAs, and tRNAs. These small RNA populations contained more diverse small RNAs with higher reads in superior spikelets than in inferior spikelets at all the grain-filling stages (Figure 2C and Additional file 1).

### Most miRNAs expressed higher in superior than in inferior spikelets and a few miRNAs showed the opposite

To understand how miRNAs were expressed in superior and inferior spikelets during the grain filling process, the abundance of each miRNA from the same libraries was normalized to transcripts per million (TPM) as performed in our previous publication [4]. The clean reads of small RNAs were mapped to miRNA precursors in miRBase release 17.0 and altogether 457 known miRNAs were identified in the ten libraries (Additional file 2). To extract meaningful data for further analysis, only miRNAs whose expressions were higher than 10 TPM at least in one of the datasets were selected. Following this criterion, a total of 160 known miRNAs were chosen for their expressional analyses (Additional file 3). We found that 141 miRNAs were differentially expressed between superior and inferior spikelets at least in one of the same filling stages, and most of them were more abundant in superior than inferior spikelets at early and middle grain filling stages (10–27 DAF).

**Figure 3:**
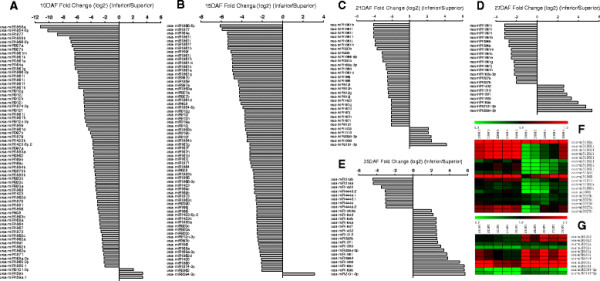
**Differentially expressed miRNAs between superior and inferior spikelets during rice grain filling. (A-E)**. Bar graphs reflecting expressional fold changes of selected miRNAs between superior and inferior spikelets at 10DAF **(A)**, 15DAF **(B)**, 21DAF **(C)**, 27DAF **(D)**, and 35DAF **(E)**, respectively. Expressional fold changes were conducted following detailed descriptions in methods and materials. Values in fold changes and their ranges are indicated by the ratio of miRNA expressions of inferior over that of superior spikelets from less than (−) to more than (+) the equal expression (0) between inferior and superior spikelets. Only miRNAs whose expressions were higher than 10 TPM in each of the ten databases were selected for calculating their expressional fold changes and only the absolute value changes higher than 2 were listed in the figures. **(F-G)**. Heat maps showing the expression levels [log2(normalized expression)] of selected miRNAs that were highly expressed in superior spikelets **(F)** and inferior spikelets **(G)** at different rice grain filling stages (P < 0.05, n = 5, two-tailed paired t-test).

In addition, the largest difference between superior and inferior spikelets was found at 15 DAF, and among the 109 differentially expressed miRNAs, 103 (~94.50%) miRNAs expressed higher in superior spikelets (Figure 3A-E; Additional file 4). Furthermore, among the 160 higher expressed miRNAs, 19 miRNAs (~11.88%), such as miR168a, miR1861b,d,f,h-j,l, miR1864, miR1868, miR1873, miR1883a,b, miR408, miiR815b-d, and miR827a,b, were expressed noticeably higher in superior than inferior spikelets at all stages in rice grain filling (P < 0.05, n = 5, two-tailed paired t-test; Figure 3F). In contrast, only 11 out of the 160 miRNAs (~6.88%), miR1318, miR1432, miR162a,b, miR164a,b,f, miR166k,l, miR2094-3p, and miR2101-3p, were more abundantly expressed in inferior spikelets at the similar grain-filling stage during rice grain filling (P < 0.05, n = 5, two-tailed paired t-test; Figure 3G).

### Targets of the differentially expressed known miRNAs related to rice grain filling

**Figure 4:**
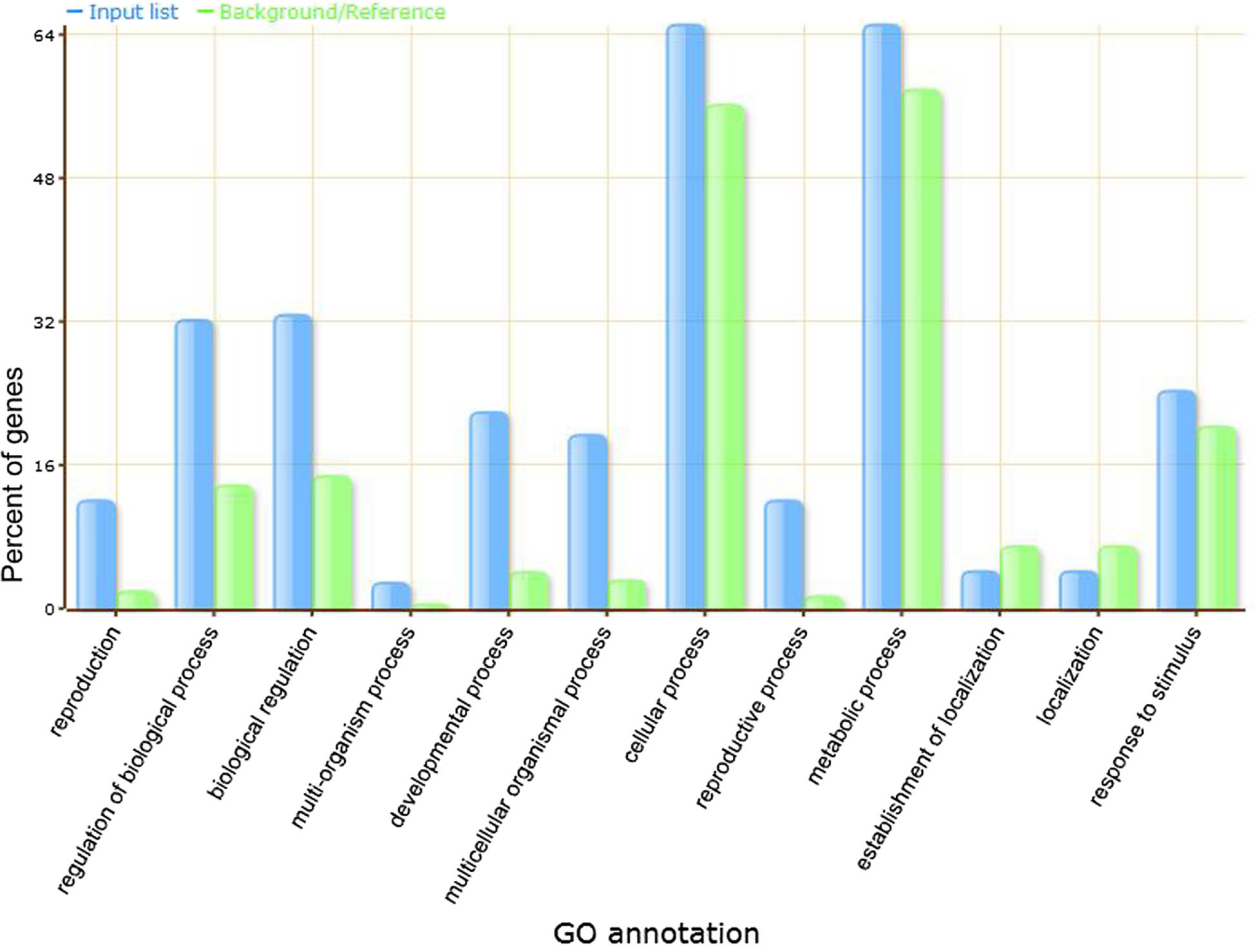
**Distributions of differentially expressed miRNA target genes and their functional categories determined by GO analyses.** The x axis represents categories of biological functions in different terms. The y axis is the percentage of genes involved in a certain function. The blue bars represent the percentage of input genes, targets of the differentially expressed miRNAs between inferior and superior spikelets with specific functions. The green bars represent the percentage of the control genomic genes that are involved in the specific function of the same category. If the percentage of the special functional category of input genes’ were higher or lower than that in the control genomic genes indicate this functional category may regulated by the differential expressed miRNA targets.

Plant miRNAs recognize and bind imperfectly to their target mRNAs through base-pairing, leading to mostly mRNA cleavage or, to some extent, translational repression at the post-transcriptional levels [29]. Genome-wide miRNA-directed target mRNA cleavage can be identified by a high-throughput sequencing method known as degradome analysis or parallel analysis of RNA ends. This method was successfully used in rice to identify rice miRNA’s targets [30]–[32]. Recently, the identified targets of rice miRNAs using such a technology have been summarized and reanalyzed through SeqTar based on a new algorithm [33], providing a useful platform for us to analyze potential miRNA targets during rice grain filling process. To analyze the function of these differentially expressed miRNAs in grain filling, their targets identified by SeqTar were collected and used for GO enrichment analysis. As a result, 221 target mRNAs were found and submitted to AgriGO at the published website for further analysis [34] (Additional file 5). After the GO biological process enrichment analysis in contrast to their expression backgrounds/references, these miRNA target genes were functionally categorized into the following specific biological processes with or without significant differences from their respective backgrounds/references (Figure 4):

1) miRNA target genes were largely from “cellular process” (65.24%), “metabolic process” (65.24%), and “response to stimulus” (24.39%) with small differences from their backgrounds/references; (2) Small portions of miRNA target genes were functioning in “localization” (4.27%) or the “establishment of localization” (4.27%) with no differences from their backgrounds/references; and finally but not least (3) A fairly large amount of important miRNA target genes were functioning in “reproduction” (12.20%), “biological regulation” (32.93%), “regulation of biological process” (34.42%), “developmental process” (21.95%), “reproductive process” (12.20%), and the so-called “multicellular organismal process”(19.51%), “multi-organism process” (3.05%) with significant differences from their backgrounds/references. Among these important miRNA target genes that had significant expressional differences from their backgrounds/references, genes functioning in “reproduction”, “biological regulation”, “regulation of biological process”, “developmental process”, and “reproductive process” are extremely important in relation to the rice grain development and grain filling process. Relations of some representative key regulatory genes and their corresponding miRNA regulators during the grain filling processes of both superior and inferior spikelets were further analyzed below.

**Figure 5:**
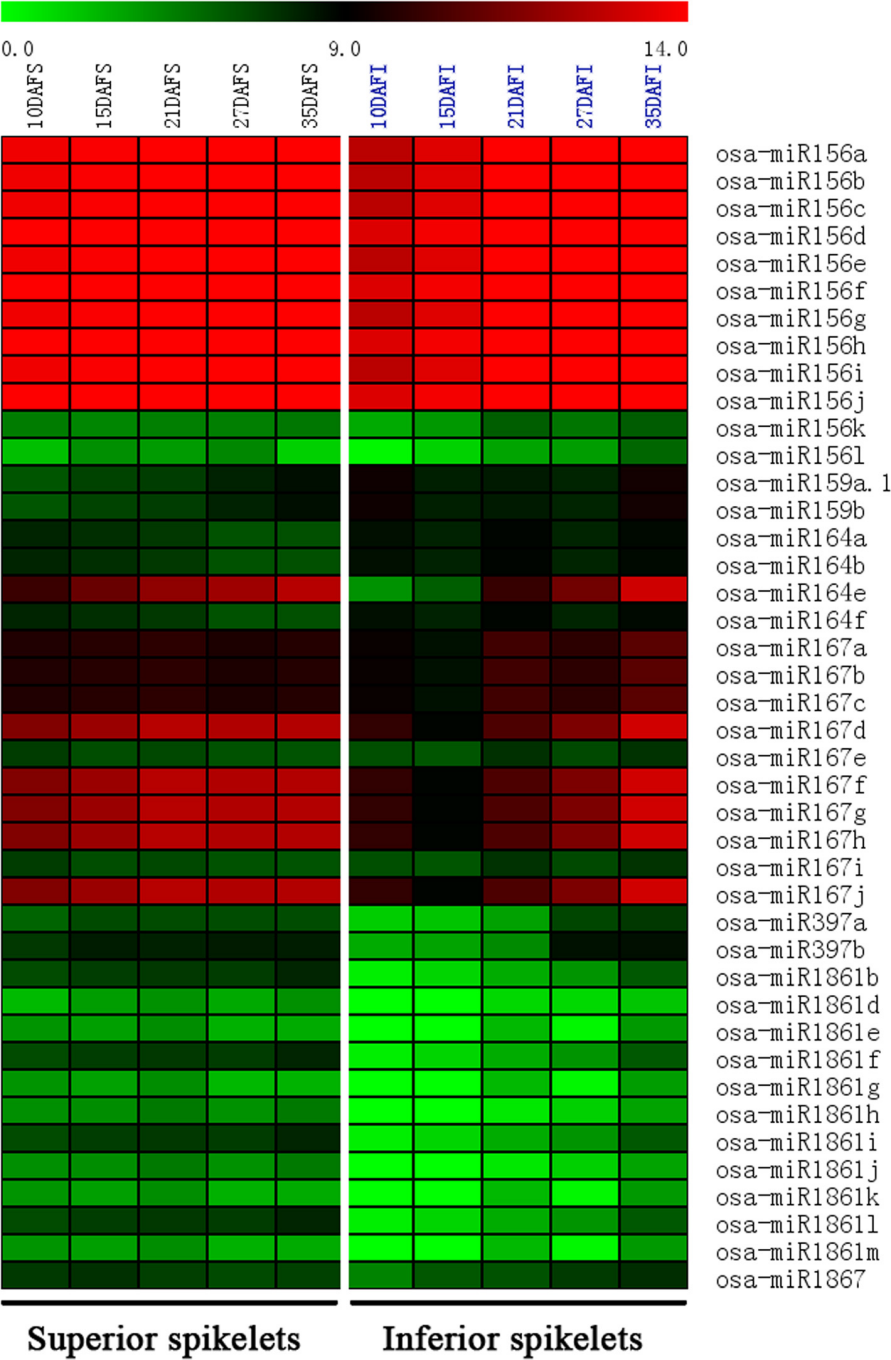
**Expression analysis of specific miRNA family members that potentially contribute to the differential rice grain filling between superior and inferior spikelets.** Heatmap shows the expressional levels [log2(normalized miRNA’ expression)] of different miRNA family members including miR156, miR159, miR164, miR167, miR397, miR1861, and miR1867 families in superior (S, on left part of the map) and inferior (I, on right part of the map) spikelets at different rice grain filling stages.

**Figure 6:**
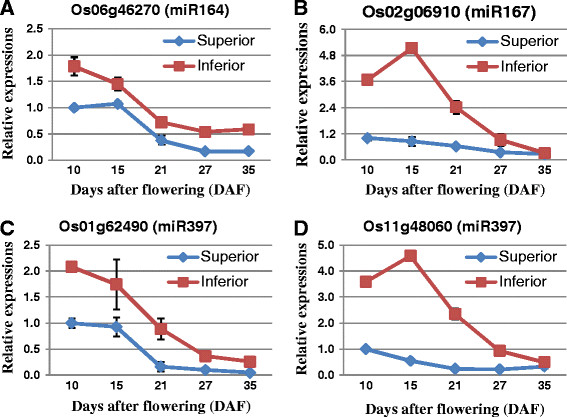
**The expressional patterns of a few auxin-related key miRNA target genes and their differential expressions between superior and inferior spikelets.** Gene expression levels were assayed by qRT-PCR and indicated in lines. Three miRNA target genes, miR164 target gene Os06g46270 **(A)**, miR167 target gene Os02g06910 **(B)**, and miR397 target genes Os01g62490 **(C)** and Os11g48060 **(D)** were measured for their expressions at 10, 15, 21, 27 and 35DAF in superior (blue) and inferior (red) spikelets.

In general, the expressions of miRNAs and their targets show negative correlations if simple regulations exist between them [16],[21]. To identify the expression patterns of key miRNAs and their targets that are related to rice grain filling, we selected targets of three highly expressed miRNAs (miR164, miR167, and miR397), which may have crucial roles in rice grain filling, for further study by qRT-PCR. Specially, miR164 and miR167 are auxin-related miRNAs (auxin-miRs) that determine the cellular levels of free auxin through down-regulating their target NAC and ARF family transcript factors [21],[35],[36]. These auxin-miRs may play a role in controlling rice grain filling. miR397 via targeting a transcript encoding a laccase-like protein, positively regulates rice grain size and panicle branching [17]. Indeed, the relative expressions of these miRNA target genes had a strong but simple negative correlation with the levels of their corresponding miRNAs in both the superior and inferior spikelets during the rice grain filling processes (Figures 5 and 6).

### Novel grain-filling related miRNAs and their predicted targets identified in superior and inferior spikelets

To identify novel miRNAs in superior and inferior spikelets during rice grain filling, miRNA candidates were first collected from MIREAP and then determined to be novel miRNAs if they have not been reported and their corresponding miRNA*s were also identified in one of our library [37] or the potential miRNAs were detected in more than half of the ten libraries [28]. Among all the candidate novel miRNAs, two (miRn1 and miRn2) were highly expressed (>50TPM) in at least one of our dataset and were thus included as novel miRNAs. Following these criteria, altogether, 13 novel miRNAs were identified to have perfect stem-loop secondary structures but have never been reported (Table 1, Additional file 6). Among these novel miRNAs, most showed differential expressions in a panicle-location and/or developmental-dependent manner. For instance, miRn2 was identified to be highly expressed only during the early and middle grain filling stages in inferior spikelets (10–21 DAFI), but not expressed in superior spikelets or in inferior spikelets at later grain filling stages (Table 1). Furthermore, the expressions of three newly identified miRNAs were validated by stem-loop qRT-PCR, and the results were consistent with the data we collected from high throughput sequencing (Additional file 7). Finally, we predicted the target genes for these 13 new miRNAs based on psRNATarget proposed by Dai et al. [38]. Altogether, 68 candidate target genes were predicted for the 13 novel miRNAs except miRn2 (Additional file 8). Two target genes of miRn6 were selected for validation by RNA ligase–mediated 5’-RACE because these two targets can be identified in the degradome database PsRobot [39]. The miRNA binding sites of these two targets were located on the 3’ or 5’ untranslated regions (UTRs), but the cleavage sites were somehow immediately downstream or upstream of the miRNA binding sites (Additional file 9). Two other target genes of miRn6 were also observed in the degradome database termed starBase (Additional file 10) [40], suggesting that this newly identified miRNA may target their predicted genes for regulations. Nevertheless, these non-canonical cleavage sites have never been reported previously, suggesting either an unidentified mechanism by which the target mRNA was processed or that these putative targets were just random degradated and they were not the miRNA *bona fide* targets.

**Table 1:** Newly identified miRNAs in superior and inferior spikelets during rice grain filling

**miRNA ID**	**Sequence**	**Length**	**10DAFS**	**15DAFS**	**21DAFS**	**27DAFS**	**35DAFS**	**10DAFI**	**15DAFI**	**21DAFI**	**27DAFI**	**35DAFI**	**Evidence**^**#**^
miRn1	TCTTCGATAAGAATGCTGGCA	21	0	0	0	0	0	1.03	0	181.5	0	0	H
miRn2	TGATGTGTAGCACAATGCGGCTT	23	0	0	0	0	0	254.34	176.41	93.89	0	0	H
miRn3	TTGAGACGTAGAGGATAAGGT	21	0	0	0	1.29	1.31	0	1.79	1.58	0	2.31	F
miRn4	TTGGCAACGGACGCGATGGT	20	1.97	0.9	0	0	0	3.27	2.47	2.16	2.2	0.58	F
miRn5	TTTTGCTCAAGACCGCGCAAC	21	0.91	0.45	0	0	0	2.84	1.45	1.78	0.34	0	F
miRn6	TAAAGGAAGAAGAGAGAGAGT	21	0.7	0.6	0	0	0	3.27	0.6	1.14	0.56	0.72	F
miRn7	TGGATACTGGTAGAGGCGCCGCT	23	0	0	0	0	0	0	0	0	0	1.3	*
miRn8	TCCGACGCGAACTGGATGAGGCC	23	0	0	0	0	0	0.6	0	0	0	0	*
miRn9	TTCTGCTTGTGTATCGTCGCC	21	0	0	0	0	0	1.55	0	0.63	0	0	*
miRn10	AAGTGTGTAATGTTGAACGGA	21	0	0	0	0	0	1.03	0	0	0	0	*
miRn11	TGGTGAGCCACTGGGATGAGGATG	24	0	0	0	0	0	0	0	1.01	0	0	*
miRn12	TTTGAGATCTGGTGAGAATGTA	22	0	0	0	0	0	0	0	0.89	0	0	*
miRn13	AAGGGGCGCTTACTGAGAGTTCT	23	0	0.38	0	0	0	0.43	0	0	0.28	0.36	*

^#^Evidence of identified miRNA, ‘*’ indicates the detection of the corresponding miRNA. ‘F’ indicates that the miRNA were founded at least in five of the ten libraries. ‘H’ represents the abundance of the miRNA higher than 50 TPM in one of the ten databases.

## Discussion

Despite as many as 547 miRNAs and one miRNA* and their expressions at certain specific developmental stages have been documented for *Oryza sativa* in the miRBase release 17.0, knowledge of miRNAs dynamics in grain filling and grain development specifically in superior and inferior spikelets is lacking. In this study, high-throughput sequencing technology was employed to gather rich sequence reads information which makes investigation of the miRNA dynamics possible in grain filling during the development of superior and inferior spikelets.

In plants, the majority of small RNAs are 21-24nt in length. Among them, most miRNAs are 20-22nt, while most siRNAs are either 21-nt or 24-nt in length [41]. In addition, 23-nt small RNAs were also reported and might represent a new kind of functional small RNA [42]. Using high-throughput sequencing technology, over 10 million clean reads were obtained from superior and inferior spikelets at five different developmental stages during rice grain filling process. Unlike the rice leaf and tricellular pollen tissues where the 21-nt small RNAs were the major reported small RNA population [28], filling or developing grains contained the most abundant 24-nt small RNAs by sequencing of ten small RNA libraries constructed from different stages of growing grains. Furthermore, the expressions of these 24-nt siRNAs were extremely dynamic in a developmental stage-dependent manner in rice grain filling, indicating that 24-nt siRNAs may also play a key role in the formation of distinct grain filling patterns between superior and inferior spikelets, because these 24-nt siRNAs are related to certain important rice KEGG pathways, such as the starch and sucrose biosynthesis, as discussed in our previous publication [43].

### Differential expression patterns of miRNAs may determine the differential grain filling patterns of superior and inferior spikelets or vice versa

260 known miRNAs and 13 novel miRNAs were identified in at least one of the ten small RNA libraries from different developmental filling grains in rice. Expressions of miRNAs in a developmental stage-dependent and/or tissue-specific manner are the hallmarks of small RNAs found in plant development [28],[29],[42]. Grain filling process has no exceptions. By massively parallel sequencing, we detected almost all of the miRNAs that were expressed higher than 10 TPM had their unique expressional changes throughout different developmental stages of the rice grain filling process. The majority of these miRNAs increased gradually as the grain filling progressed in both superior [16] and inferior spikelets (cluster I_10_, Additional file 11). These findings expanded the previous understanding on expressions of many miRNAs focused on from relatively earlier stages of grain development such that 1–10 DAF grains had more expressions at the second half period of such short timeframe than the first half time window [19],[20] or other fragmented study of miRNA dynamics in Indica rice grain development [18], to a more systematic study of miRNAs over the entire rice grain developmental process.

Specifically, our study showed that the majority of miRNAs in superior spikelets expressed higher at the initial and middle stage (10 and 27DAF), and peaked at 15DAF, whereas in the inferior spikelets, most miRNAs had higher expression levels at the later stage (35DAF) (Figure 3, Additional file 4). Since the overall expressions of miRNA target genes were in generally negative correlation with overall expression levels of miRNAs during grain filling process, this observation explained why the previously reported 1058 out of 1261 genes were expressed over two folds lower in superior spikelets than in inferior spikelets at 9 days post-anthesis in rice [44]. Thus, simple negative regulations by miRNAs were dominant in rice grain filling by our observation. So far, we could not conclude that the differential expression patterns between superior and inferior spikelets determine the difference of grain qualities between the two types of grains in rice. It could be just the opposite that their developmental differences determine the differential expressions of miRNAs in rice.

### Importance of individual miRNAs in grain filling activities of superior and inferior spikelets

In animals, individual miRNAs have multiple targets and a single protein-coding gene is normally regulated by multiple miRNAs. In contrast, plant miRNAs have narrow but important target gene spectrums. Thus the importance of individual miRNAs is often visible when their expressions are altered. In the case of miR156, for example, *OsSPLs* are its targets that are under strict control by miR156 in the young panicles due to a predominant expression of miR156 in panicles. Over-expression of miR156 in rice resulted in severe dwarfism, significant reduction of panicle size, and flowering delay [45]. More specifically, *OsSPL14*, targeted by miR156, controls shoot branching development at the vegetative growth stage, which forms the basis for generating ideal rice plant with increased lodging resistance and enhanced grain yield [23],[24]. *OsSPL16*, another miR156 target, encodes a protein that positively regulates cell proliferation. Over expressing *OsSPL16* promoted cell division and grain filling and hence increased grain width and rice yield [25]. In our study, miR156 not only highly expressed in superior and inferior spikelets during rice grain filling, but also showed a dynamic expressional change along grain filling progresses. Specifically, miR156 is expressed higher at early grain filling stage (10–15 DAF), but lower at middle and later grain filling stage in superior than inferior spikelets (Figure 5, Additional file 2 and Additional file 3). The differential expression patterns of miR156 have a strong correlation with the difference of grain filling rate (Figure 1A) between superior and inferior spikelets, suggesting a potential contribution of miR156 to the grain filling difference between superior and inferior spikelets.

As an individual miRNA, miR397 was highly expressed in both superior and inferior spikelets during the early and middle grain filling stage, and decreased continuously along the whole grain filling period. Overall, miR397 was much lower expressed in inferior than superior spikelets, especially during the early and middle grain filling processes (Figure 5, and Additional file 2 and Additional file 3). It has been reported that miR397 positively controls the rice grain size through down-regulating its target, *OsLAC*, a laccase-like protein that may control plant sensitivity to brassinosteroid [17]. Brassinosteroid hormone levels can be regulated by C-22 hydroxylases in rice. Over-expressing C-22 hydroxylases in rice will enhance the tiller and seed numbers and grain weight, especially the weight of the seeds at the bases of the spikelets, increased in C-22 hydroxylases over-expressed transgenic rice [46]. We found that lower grain weight of inferior spikelets might result from the lower expression of miR397 during the grain filling of inferior spikelets (Figure 5, Additional file 2 and Additional file 3).

MiR159, another individual miRNA, induced by ABA, has a role in controlling transcript levels of two MYB factors during Arabidopsis seed germination [47]. Although both superior and inferior spikelets contained a good amount of miR159, this miRNA was expressed higher in inferior spikelets than in superior spikelets in our study (Figure 5, Additional file 2 and Additional file 3). Previous researches demonstrated that cell-division, grain-filling rates, as well as starch accumulation in rice grains were significantly and positively correlated with ABA contents [48],[49]. Application of ABA at early grain-filling stage can significantly enhance endosperm cell division rate, so as to increase cell number, and promote grain-filling rate and the final grain weight of inferior spikelets [48],[50]. Indeed, it has been reported that miR159ab double mutant has pleiotropic morphological defects, including altered growth habit, curled leaves, and most importantly, small siliques and small seeds in Arabidopsis [51].

Rice seed development is actually a process for starch deposition, due to starch accounts for 80 ~ 90% of the final weight of brown rice. As one of the key enzymes involved in the starch synthesis pathways, activity of starch synthase was shown to be significantly and positively correlated with rice grain filling rate [5]. Interestingly, such a key enzyme has been found to be regulated by miR1867 as revealed by the 5’-RACE and degradome analysis [33]. Our data showed that miR1867 was highly but differentially expressed in superior and inferior spikelets (Figure 5, Additional file 2 and Additional file 3). Additionally, miR1861 has been demonstrated to target genes that encode beta-amylase and starch-binding domain containing proteins and thus may also play curial roles during rice seed development [16],[33]. Interestingly, miR1861 expressed much lower in inferior spikelets than in superior ones during all the grain filling stages (Figure 5, Additional file 2 and Additional file 3).

### Roles of miRNAs in auxin homeostasis

Auxin homeostasis and signal transduction related miRNAs, including miR160 [52], miR164 [35], miR167 [53],[54], miR390 [55],[56], and miR393 [57],[58], have been reported in Arabidopsis. These miRNAs belong to highly conserved miRNAs between rice and Arabidopsis and among different plant species. While miR160, miR390, and miR393 expressed at comparatively low levels during rice grain filling in our study, miR167 and miR164 were very abundant in both superior and inferior spikelets (Additional file 2). By comparing their expressions between superior and inferior spikelets, we found miR164 and miR167 were higher expressed in superior spikelets at all grain filling stages except 35 DAF. Specifically, miR164e expressed 80.3 and 42.4 times higher in superior than in inferior spikelets at 10 and 15 DAF, respectively (Figure 5, Additional file 2 and Additional file 3). Similarly, miR167d,f-h,j were expressed 9.4 times more in superior spikelets than the inferior spikelets at 15DAF (Figure 5, Additional file 2 and Additional file 3). Furthermore, the expressions of both miR164 and miR167 in superior spikelets were gradually increased during rice grain filling. In contrast, in inferior spikelets although the expression of miR164 was also increased along with grain filling process, miR167 first slightly decreased at the initial grain filling stage and then was up-regulated. These tiny differential expressions in miRNAs between superior and inferior spikelets may impose a big impact on the differential grain filling behaviors between the two types of spikelets since the tiny changes of these miRNAs-controlled auxin homeostasis were enough to change the grain filling activities. Indeed, miR167-ARF8-OsGH3.2 regulatory node was observed to play a key role in tuning cellular free auxin contents in rice callus development [36]. Similar regulations of auxin content may exist in grain filling process.

**Figure 7:**
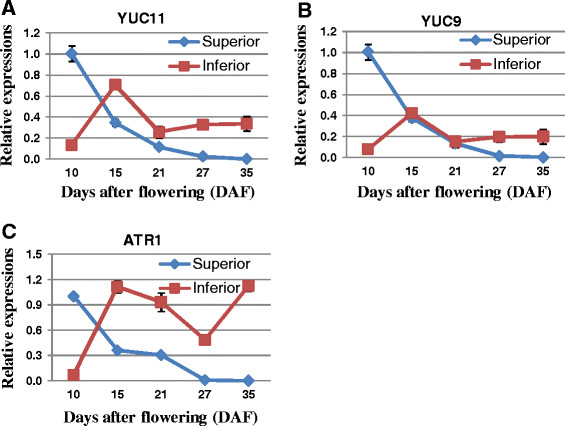
**The expression patterns of the auxin related genes during rice grain filling.** Line charts show the expressional levels of auxin related genes during rice grain filling processes in both superior and inferior spikelets. The expression levels of *YUC11***(A)**, *YUC9***(B)** and *ATR1***(C)**, three auxin metabolism related genes, were assayed by qRT-PCR method.

**Figure 8:**
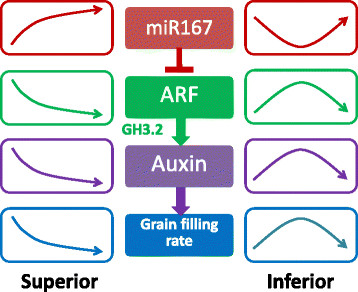
**A Model for role of miR167 and its regulatory mechanism in rice grain filling.** The trend of the arrow represents the level of gene expression and the level of grain filling rate dynamic changes with superior and inferior spikelets filling. In this model, grain filling rates are positively correlated with the auxin contents which are positively regulated by the target of miR167 through OsGH3.2 (encoding a rice IAA-conjugating enzyme), an Arabidopsis DFL1 homolog gene in rice.

Auxin content may indeed play a key role in grain filling. It has been reported that the expression of putative IAA biosynthesis genes, *OsYUC9*, *OsYUC11*, and *OsTAR1* were strongly correlated with IAA content in developing rice seeds, and may influence rice grain filling by regulating starch deposition [59]. Therefore, expression patterns of these genes in spikelets during rice grain filling were tested by qRT-PCR method in our study, and the results indicated that these genes were gradually decreased in superior spikelets. In inferior spikelets, however, they were first up-regulated until 15 DAF, and then reduced in the middle of grain filling process. While *OsYUC9* and *OsYUC11* remained almost unchanged since 21 DAF, *OsTAR1* kept decreasing until 27DAF and then increased dramatically until the end of grain filling (Figure 7). Overall, our data showed that the expressions of these three genes were negatively correlated with the levels of miR167, while positively correlated with IAA content in superior and inferior spikelets during rice grain filling processes [60]. IAA content in developing rice seeds was positively and significantly correlated with the rates of endosperm cell division and grain filling. Therefore, differential accumulation of IAA, or differential expression of auxin-related miRNAs, such as miR167 and miR164, in grains may be a reason for poor grain filling and less final grain weight of inferior spikelets [60] (Figures 5, 6, 7 and 8).

### Mechanisms for synchronous grain filling activities in superior and inferior spikelets

While many studies over-stated roles of individual miRNAs in development, study of hundreds of miRNAs in relation to thousands of their target genes in dynamics would place us in a good stead in fully understanding of the mechanisms underlying the synchronous grain filling activities in grain development. For example, miR156 was revealed to play a key role in the establishment of rice architecture and grain size [23]–[25],[45]. miR167 is a key determiner of rice agronomic and yield characters [22],[36]. miR393 is an important player in regulating inclination of flag leaves and stress response [26],[61]. Most recently, miR397 was determined to be a key factor of modulating rice panicle branching and grain size [17]. All these miRNAs function individually by targeting and modulating the expressions of key transcription factors that determine agronomical important traits during rice development. Our study indicates that these miRNAs may function as cohorts coordinately in rice grain filling. It would be interesting to see that a change in one such miRNA or its target could alter the changes of not only specific traits but also the changes of other miRNAs and their targets in dynamic regulations.

## Conclusions

In summary, our results together with data published by other laboratories suggest that differentially expressed miRNAs, especially those that expressed differently at early grain filling stage and that are involved in the hormone homeostasis and starch deposition, etc., might or at least partially account for, the asynchronous and asymmetric filling patterns during rice grain filling and the formation of different final grain weights between superior and inferior spikelets. Future studies will focus on roles of multiple miRNAs, such as auxin related miRNAs, in the discovery process of mechanisms for poor seed filling in rice.

## Methods

### Rice cultivation, sampling, dry weights and grain-filling rate determination

Oryza sativa spp. japonica cv. Xinfeng 2 seeds were grown under stress-free condition at a research farm of Henan Agricultural University, Henan Province, China (34°53’ N, 113°35’ E, 94m altitude). Superior and inferior spikelets were sampled at 5-day intervals from 5 day after flowering (DAF) to 45DAF based on the method used previously [6]. Sample volume equals to 35 grains per sample at each sampling time point. Totally, nine sets of samples were obtained for measuring the dry weight of superior and inferior spikelets, respectively. To determine the dry weight, the samples were firstly fast dried at 105°C for 30 min, and then were kept at 80°C until constant weights were reached, which indicates a complete dryness of the grains. The grain filling rates in both superior and inferior spikelets at different time points can be determined by filling dry grain weight values into the followed equations:

(1) $$Y=K/\left(1+ae^{-\mathrm{bt}}\right)$$

The grain filling rate (V) was calculated by the derivative of Equation 1:

(2) $$V=\mathrm{Kab}e^{-\mathrm{bt}}/{\left(1+ae^{-\mathrm{bt}}\right)}^{2}$$

where Y is the average weight per grain (mg), t is number of days after flowering, K, a and b are coefficients determined from regression.

### RNA sampling, sequencing and raw deep sequencing data analyses

The dehusked immature superior and inferior spikelets at 10DAF, 15DAF, 21DAF, 27DAF, and 35DAF were our target materials for total RNA isolation by TRIzol reagent (Invitrogen, Carlsbad, CA, USA) following the manufacturer’s instructions. Extracted total RNAs were further assayed for quality by Agilent 2100 bioanalyzer. For the removal of possible DNA residuals, RNase-free DNase I was applied for 30 min at 37°C. Beijing Genomics Institute (Shenzhen, China) carried out the followed library construction and sequencing by using our RNA samples.

The adapter sequences and low quality sequences were removed from the raw data, and the length distributions of the clean reads were plotted. The resulted sequences from the ten libraries were then aligned to the rice genome by using SOAP [27]. They were further classified into tRNA, rRNA, small nucleolar RNA (snoRNA), and small nuclear RNA (snRNA) by referring to the NCBI nucleotide database and Rfam RNA family database [62]. For known miRNA identification, total small RNA clean reads were aligned to miRNA precursor sequences from rice miRNA database (miRBase 17.0).

To compare the expressional patterns and the differentially expressed miRNAs between superior and inferior spikelets at the same time point during grain filling, the abundance of each miRNAs in the ten libraries were normalized to transcripts per million (TPM) as previously described [28]. After normalization, we set the expression to 0.01 TPM for those miRNAs that were not expressed. As for those miRNAs that expressed less than 10 TPM in all of the sequencing libraries, their differential expressions were ignored.

### Calculation of the fold-changes and P-values from the normalized expressions

We define a character R indicating the ratio between the normalized level of a miRNA in inferior spikelets to the same miRNA normalized expressional level in superior spikelets at the same period during rice grain filling. And then the fold change of the same miRNA at the same time point between inferior and superior spikelets was equal to the binary logarithm of R.

To calculate the P-value, we can use the formula below:

(3) $$p\left(x|y\right)=\left(\frac{N_{2}}{N_{1}}\right)\frac{\left(x+y\right)!}{x!y!{\left(1+\frac{N_{2}}{N_{1}}\right)}^{\left(x+y+1\right)}}\begin{array}{c} C\left(y\leq y_{\text{min}}|x\right)=\sum_{y=0}^{y\leq y_{\text{min}}}p\left(y|x\right)\\ C\left(y\geq y_{\text{max}}|x\right)=\sum_{y\geq y_{\text{min}}}^{\infty }p\left(y|x\right) \end{array}$$

In the formula, N1 and N2 stand for the total number of clean tags in superior and inferior spikelets, respectively, in the same grain filling time. X is the number of surveyed miRNAs, and y is the number of homologous miRNAs in superior spikelets.

A miRNA was considered to be differentially expressed if the fold-change is either bigger than 1 or smaller than −1, in the prerequisite that the corresponding P-value is from 0.01 to 0.05, while when in the prerequisite that the P-value is smaller than 0.01, the miRNA was treated as significantly differentially expressed between superior and inferior spikelets.

### Identification of novel miRNAs and target gene prediction

For the clean reads that were unable to be aligned to NCBI nucleotide database, Rfam RNA family database or miRBase, were subjected to ‘MIREAP’ (http://sourceforge.net/projects/mireap/) to identify the novel miRNA candidates. For those potential miRNAs who have their corresponding miRNA* sequences present in at least one library, or whose mature sequences could be detected from more than half of our libraries, or who have more than 50 TPM mature sequence reads in at least one library, were finally defined as novel miRNAs.

The psRNATarget program (http://plantgrn.noble.org/psRNATarget/) with default parameters was used to predict miRNAs’ potential targets. The sequences of newly identified miRNAs were used as input sequences. The *Oryza sativa* TIGR genome cDNA OSA1 Release 5 (OSA1R5) was used as the genomic library for the target search.

### Gene ontology (GO) analysis

GO analysis enable us to see the targeted genes’ functional distributions or emphases of the differentially expressed miRNAs in inferior and superior spikelets. The GO analysis tool is on this website: http://bioinfo.cau.edu.cn/agriGO/analysis.php[34]. We used TIGR locus version 091211 as background or reference. The background can give us an idea on the percentage of genes involved in a certain function, against the whole genome. The input will be our identified targeted genes of the differentially expressed miRNAs between superior and inferior spikelets. After running, we can know the percentage of those differentially expressed miRNAs’ targeted genes that involved in a certain function. By comparing the percentage of input and that from the background, we can find the functional emphases of those miRNAs’ targeted genes.

### Quantitative real-time RT-PCR (qRT-PCR) and stem-loop qRT-PCR

Briefly, 1 μg of total RNAs pre-treated with DNase I (Promega), was reverse-transcribed with the help of reverse transcriptase (Promega) to generate cDNA. 5 μl of the 1:20 diluted cDNA was used as template in a 20 μl PCR system, mixed with the right amount of SYBR green reaction solution (SYBR® Green QRT-PCR Master Mix; Toyobo). The qPCR system was firstly pre-incubated at 95°C for 5min, and then went to the 40 cycles including denaturation at 95°C for 15s, annealing at 60°C for 15s, and extension at 72°C for 32s. BioRad iQ5 sequence detection system is the equipment we used (BioRad, USA). Stem-loop qRT-PCR used for miRNA expression pattern analysis was performed as we described previously [4]. For normalization in brief, the copy numbers of the miRNAs in inferior spikelets were firstly normalized by comparing with rice 5.8S rRNA level following the previously published method [21], and then the expression level of the miRNAs were further normalized by comparing with their corresponding expressions in superior spikelets at 10 DAF, which were initially set to 1.0. All gene-specific primers involved in this experiment are listed in Additional file 12.

### RNA ligase–mediated 5’-RACE

To conduct the RNA ligase–mediated 5’-RACE, 1 μg total RNAs from equal mixture of superior and inferior spikelets at 10DAF, 15DAF, 21DAF, 27DAF, and 35DAF was ligated to a 5’-RACE RNA adapter without calf intestine alkaline phosphatase treatment using 5′-Full RACE Kit (Takara), followed by a reverse transcription reaction. Then 1 μl of reverse transcription product was used as template to amplify the 5’ end of the potential target by using the outer 5’ RNA adaptor primer supplied in the kit and outer 3’ gene-specific primer. Thirty more cycles of PCR were further carried out using the inner 5’ RNA adaptor primer supplied in the kit and inner 3’ gene-specific primer. The final PCR product was showed on the ethidium bromide-stained agarose gel, cloned into pGEM-T Easy vector, and at least 7 independent clones from each reaction were sequenced finally.

## Competing interests

The authors declare that they have no competing interests.

## Authors’ contributions

TP and QZ designed and preformed the experiments, analyzed the data, and wrote the manuscript. HS carried out high through-put sequencing and data analysis. QM and YZ performed the QRT-PCR. YD, JZ and JL gave technical advice and contributed to the study design. GT and QM edited the manuscript. All authors read and approved the final manuscript.

## Additional files

## Supplementary Material

Additional file 1 Summary of small RNA classes in superior and inferior spikelets during rice grain filling. Click here for file

Additional file 2 Frequency (in TPM) of known miRNAs in each library. Click here for file

Additional file 3 Known miRNAs greater than 10 TPM in at least one library. Click here for file

Additional file 4 Differential expressed miRNAs between superior and inferior spikelets during rice grain filling. Click here for file

Additional file 5 Differential expressed miRNAs' targets collected from SeqTar. Click here for file

Additional file 6 Plotting of novel miRNAs and their corresponding miRNA*s on the miRNA precursors. Click here for file

Additional file 7 Novel miRNA’s expression patterns during superior and inferior spikelets filling valid by stem-loop QRT-PCR. Click here for file

Additional file 8 Novel miRNA targets predicated by psRNATarget. Click here for file

Additional file 9 The miRn6 cleavage sites in LOC_Os02g01590 and LOC_Os11g45740. Click here for file

Additional file 10 Predicted target fragments of novel miRNAs from starBase. Click here for file

Additional file 11 Cluster of the known miRNAs (higher than 10 TPM) in inferior spikelets. Click here for file

Additional file 12 The primers used in this study. Click here for file
