# Supplementary material for: Differentially expressed microRNA cohorts in seed development may contribute to poor grain filling of inferior spikelets in rice
Source: BMC Plant Biol. 2014 Jul 23;14:196. doi: 10.1186/s12870-014-0196-4 (PMC4422267; doi:10.1186/s12870-014-0196-4)

**Additional file 6. Plotting of novel miRNAs and their corresponding miRNA*s on the miRNA precursors.** (Three paragraphs for each novel miRNA alignment. The first paragraph, miRNA name, location on the genome, length of the miRNA precursor, minimum free energy; the second paragraph, miRNA precursor, novel miRNA and it’s miRNA* are shown in red and blue, respectively; the third paragraph, predicted stem-loop structure of the novel miRNA precursor, novel miRNA and it’s miRNA* are shown in red and blue, respectively.)

> miRn1 chr01|13101:22474876:22474950:+ 75(nt) -27.30(kcal/mol)

UGCCCAUGCACGCACAGCAAGUUCUUGUACGAAGAGCUCACACAUCUUCGAUAAGAAUGCUGGCAUGUCAAAGGU


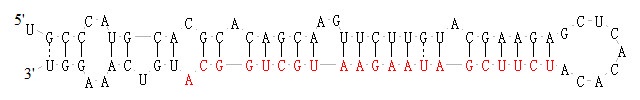


> miRn2 chr07|13107:4350994:4351074:+ 81(nt) -40.50(kcal/mol)

UGCCCCUUUGACUGGUGGCAUUGCAACCCAUCACAGGUGACUCACCUGUGAUGUGUAGCACAAUGCGGCUUGUCAAUGGUG


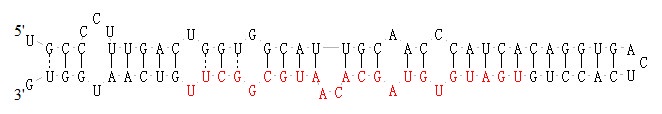


> miRn3 chr01|13101:27654880:27654977:+ 98(nt) -38.30(kcal/mol)

UUUAUCCAUUUUGAGACGUAGAGGAUAAGGUGAUUUUAAAUGUUUACUUCACACGUAGUACUGUUCAUCUUAUCCUCAAUGUCUUAAAAUAGAUAGAG


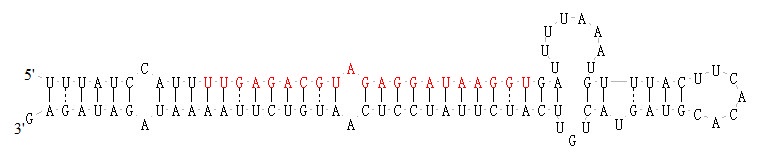


> miRn4 chr11|13111:17280609:17280712:+ 104(nt) -42.86(kcal/mol)

UAGGCGCUCCUUGGCAACGGACGCGAUGGUAAUAAAGAUUGUCCAUCGCGUCUCCGGACGCGUCUUCCCAACCAUCGCGUCUUCGGUUGCCACCCCUUCCCCAU


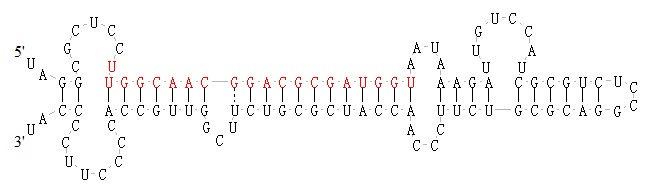


> miRn5 chr02|13102:12450906:12451130:+ 225(nt) -92.30(kcal/mol)

UUGAGAGGCGUUUUGCUCAAGACCGCGCAACGAUUCCAUAUGACACCGCAUCGUUGUCCAUGCCUAUCAUGUUCAUCCAGAGCCCCUGUUGAUCUUCAGAUAAGAGAAAAGCUCAAAAUUAAAGAAGGCUUGUUUAUUGAUCAUGUCAAGGAUAGGUAUCGACGGCGAUGCGCUGGCUAUGGCCAUGGUUGUGCGGUUCUUGCGCAAUAACGUUUUGAUCUCGAU


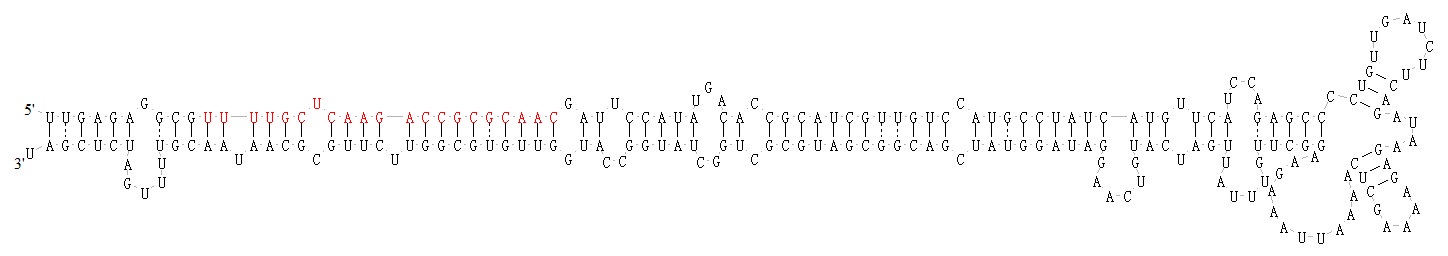


> miRn6 chr02|13102:16221920:16222050:+ 131(nt) -65.07(kcal/mol)

ACAUCGAGGCUAAAGGAAGAAGAGAGAGAGUACAGCCUUAGUUCAGAUCACAUAUGACUAUCUUUUGCGAGUCUGAUCGAUACUCUGAUGAAGGCUGUACCCUCUCUCUUCUUCUCUUAGCUUCUUGAUGC


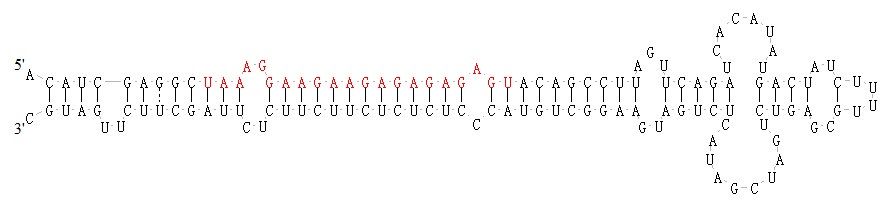


> miRn7 chr08|13108:10863846:10863916:- 71(nt) -26.90(kcal/mol)

CCUGUACGUAUGGAUACUGGUAGAGGCGCCGCUGGUUUGCGGUGCUGAUCGACGUUGGAGUACGGCGAGGA


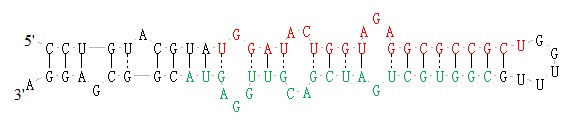


> miRn8 chr01|13101:32310273:32310346:- 74(nt) -22.90(kcal/mol)

UGUGGACUCCUCCGACGCGAACUGGAUGAGGCCGAACCCCCUGGGCAUCUUCGUGUUAAGGUCGGUGACCACGA


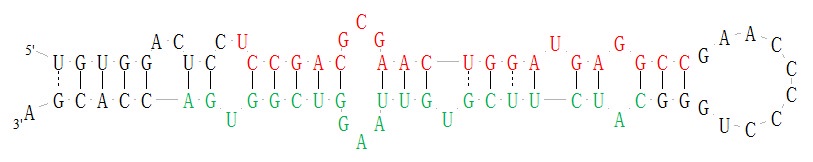


> miRn9 chr02|13102:825910:826009:+ 100(nt) -49.50(kcal/mol)

CUUGAAGUCGUUCUGCUUGUGUAUCGUCGCCUUCUGCCUCCUCCCCACCACGGGGCGCGGCGUGAUGGGCGUCGGCGGCGCGUGGCGGAGCGGCGCGUGG


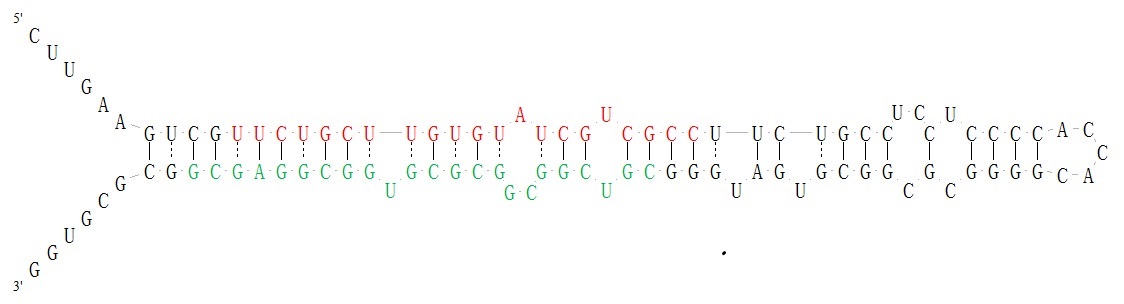


> miRn10 chr04|13104:17316251:17316345:- 95(nt) -33.00(kcal/mol)

GGCCGUGGCCAAGUGUGUAAUGUUGAACGGACUGCUUCUCUGGCCCUUGUCACGUCUUCUUGUUCAGCAUUGGAUUGGGCUUGGCGUCAGGACCA


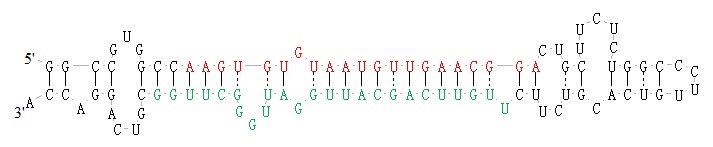


> miRn11 chr08|13108:23990659:23990914:+ 256(nt) -147.60(kcal/mol)

GAGUAAUUCGUCCUCAUCCCAAUGCUCGUCAUCUACGGUUGGAAAAAGCCAUGAACUUUAUGUGGCAUAACGGCUCACCUCUAUGGGUACACAGUGUGGAAGCGGACAAAGUCUCUAGCUCACAAAGAUGAGUGGAGAUGACUUUGUCCGCUUCCGCUCGGUCUGCCUGCAUUGGAGAACCGUUGUGCCGUGUAAAGGGCAUGAAUUCUUCCAGUUGUUGAUGAUGGUGAGCCACUGGGAUGAGGAUGAAUACUCC


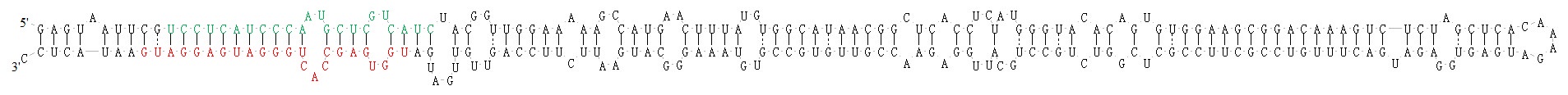


> miRn12 chr12|13112:12076962:12077093:- 132(nt) -77.92(kcal/mol)

UAGAUAUGAAAUACAUUCUCACCAGAUCUCAAAUCCAAACUCAACUUCGUUUAAGAGAAACAAAGAAGACAAAUUUCGAGUGAAUAGUGUUGAGUUUGGAUUUGAGAUCUGGUGAGAAUGUAUUUCAUAUCU


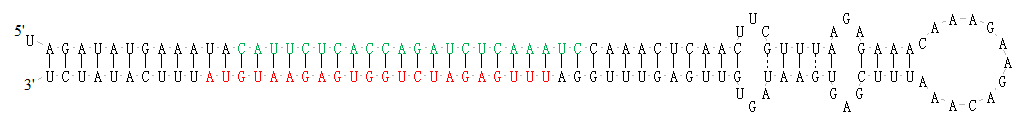


> miRn13 chr02|13102:27142265:27142413:+ 149(nt) -68.34(kcal/mol)

GGGCUUAUCAAAGGGGCGCUUACUGAGAGUUCUUUGGCAUUCUGUCCACCUCCUUGUCGAAUCCUCAGAGACAGAAAUCUCAUAUCUGUUGAUCUUGGAGGUGGGCAUACUGCCAAUGGAGCUGUGUAGGCCUCCCUUUGUAAAACCCA


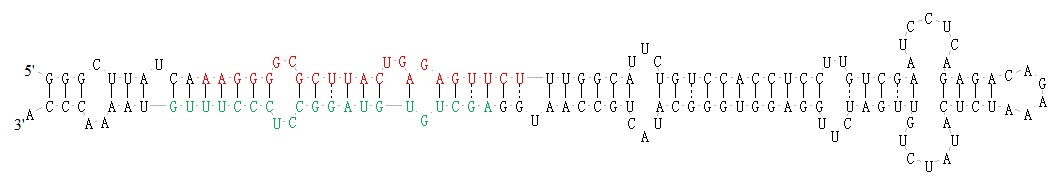

Supplement: Additional file 6 — Plotting of novel miRNAs and their corresponding miRNA*s on the miRNA precursors. [file s12870-014-0196-4-S6.docx]
