# Supplementary material for: Differentially expressed microRNA cohorts in seed development may contribute to poor grain filling of inferior spikelets in rice
Source: BMC Plant Biol. 2014 Jul 23;14:196. doi: 10.1186/s12870-014-0196-4 (PMC4422267; doi:10.1186/s12870-014-0196-4)
Supplement: Additional file 7 — Novel miRNA’s expression patterns during superior and inferior spikelets filling valid by stem-loop QRT-PCR. [file s12870-014-0196-4-S7.docx]

**Additional file 7.** Novel miRNA’s expression patterns during superior and inferior spikelets filling valid by stem-loop QRT-PCR.
