# Supplementary material for: Differentially expressed microRNA cohorts in seed development may contribute to poor grain filling of inferior spikelets in rice
Source: BMC Plant Biol. 2014 Jul 23;14:196. doi: 10.1186/s12870-014-0196-4 (PMC4422267; doi:10.1186/s12870-014-0196-4)
Supplement: Additional file 9 — The miRn6 cleavage sites in LOC_Os02g01590 and LOC_Os11g45740. [file s12870-014-0196-4-S9.docx]

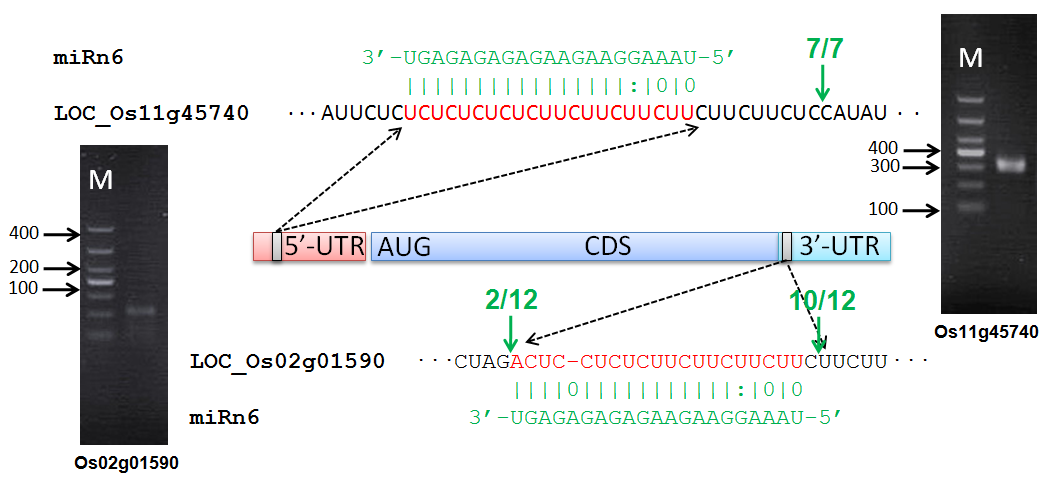


A


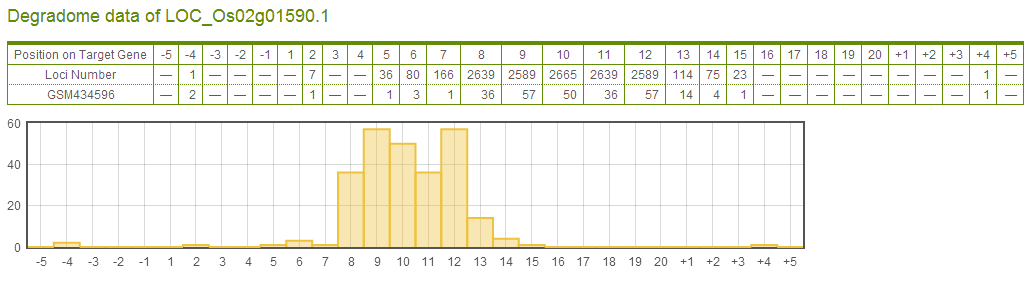

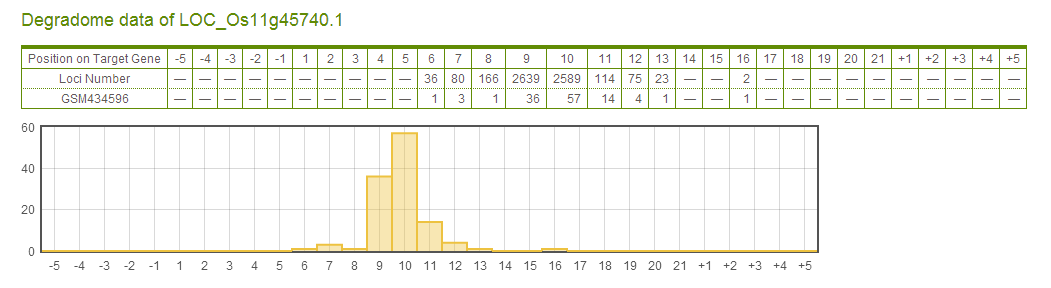


C

B

**Additional file 9. The miRn6 cleavage sites in** **Loc_Os02g01590 and Loc_Os11g45740.** (A) The miRn6 cleavage sites in Loc_Os02g01590 and Loc_Os11g45740 were identified by RNA ligase–mediated 5’-RACE in developing superior and inferior spikelets. Each nested PCR product is showed on the ethidium bromide-stained agarose gel. The frequency of clones was shown on the top of the arrow. Lane M represents a DL1000 DNA ladder. (B, C) The miRn6 cleavage sites in Loc_Os02g01590 (B) and Loc_Os11g45740 (C) were determined by degradome data deposited in PsRobot (Wu et al., Nucleic Acids Research, 2012).
