# Supplementary material for: Differentially expressed microRNA cohorts in seed development may contribute to poor grain filling of inferior spikelets in rice
Source: BMC Plant Biol. 2014 Jul 23;14:196. doi: 10.1186/s12870-014-0196-4 (PMC4422267; doi:10.1186/s12870-014-0196-4)
Supplement: Additional file 10 — Predicted target fragments of novel miRNAs from starBase. [file s12870-014-0196-4-S10.docx]

**Additional file 10.** Predicted target fragments of novel miRNAs from starBase.

| **miRNA** | **Target** | **miRNA squence** | **Degradome data** | **Cleavage tags** | | | **Penalty Score** | **Target Description** |
| --- | --- | --- | --- | --- | --- | --- | --- | --- |
|  |  |  |  | **GSM455938/rice seedling^a^** | **GSM455939/rice young panicle^a^** | **GSM476257/young inflorescences (4~6cm)^a^** |  |  |
| miRn6 | LOC_Os06g10230 | TAAAGGAAGAAGAGAGAGAGT | ACTTCTTTCTTCTTCCTTTT | 0 | 0 | 2 | 2.5 | receptor-like protein kinase 5 precursor, putative, expressed |
| miRn6 | _ | TAAAGGAAGAAGAGAGAGAGT | GCTTTCTCTTCTTCCTTTTA | 0 | 1 | 0 | 4.5 | nocoding RNA from MST |

^a^ The rice degradome samples/sources.
