# Supplementary material for: Differentially expressed microRNA cohorts in seed development may contribute to poor grain filling of inferior spikelets in rice
Source: BMC Plant Biol. 2014 Jul 23;14:196. doi: 10.1186/s12870-014-0196-4 (PMC4422267; doi:10.1186/s12870-014-0196-4)
Supplement: Additional file 12 — The primers used in this study. [file s12870-014-0196-4-S12.docx]

**Additional file 12.** The primers used in this study.

| **Primer Name** | **Sequence** |
| --- | --- |
| **qRT-PCR primer** |  |
| Os02g06910 (ARF6) F | GGAAGACCCTTTGAGATCAG |
| Os02g06910 (ARF6) R | CACAAATTCCCTGCAGATT |
| Os06g46270 (NAC21/22) F | TGCCTCCCCTCACTGACAAC |
| Os06g46270 (NAC21/22) R | CAACCATGGCCGATGTCAAC |
| Os01g62490 (AO) F | TCTACAACTGCTCTGCCAAAG |
| Os01g62490 (AO) R | CGGCGATGGAGAAGAAGA |
| Os11g48060 (Laccase) F | ACAATGCACAGATGGGTTCA |
| Os11g48060 (Laccase) R | GGCTTGGTATAGACTGCATCA |
| OsYUC9_F | CTGGCTCAAGAGTGATGACG |
| OsYUC9_R | TCCTCGTAGCTGCCGTAGAT |
| OsYUC11_F | ACTGGCTTAAGAATGGTGAGGA |
| OsYUC11_R | GCGTCATGAGCAATACCAGA |
| OsTAR1_F | GCACCATACTACTCCTCGTACCC |
| OsTAR1_R | GACGAGCTCGACGTAGGTGT |
| actin_F | GGAAGTACAGTGTCTGGATTGGAG |
| actin_F | TCTTGGCTTAGCATTCTTGGGT |
| **Stem-loop qRT-PCR primer** |  |
| miRn2_RT | CTCAACTGGTGTCGTGGAGTCGGCAATTCAGTTGAGAAGCCGCA |
| miRn2_F | ACACTCCAGCTGGGTGATGTGTAGCACAATG |
| miRn4_RT | CTCAACTGGTGTCGTGGAGTCGGCAATTCAGTTGAGACCATCGC |
| miRn4_F | ACACTCCAGCTGGGTTGGCAACGGACGC |
| miRn5_RT | CTCAACTGGTGTCGTGGAGTCGGCAATTCAGTTGAGGTTGCGCG |
| miRn5_F | ACACTCCAGCTGGGTTTTGCTCAAGACCG |
| Stem-loop_U | TGGTGTCGTGGAGTCG |
| Os5.8s rRNA_F | GCAACGGATATCTCGGCTCTCGC |
| Os5.8s rRNA_R | GGATGGCCTCGGGCGCAACT |
| **RNA ligase–mediated 5’-RACE primer** | |
| Os02g01590_Outer | AAAGAGGGAGGGAGAGAGAAACATA |
| Os02g01590_Inner | TTGACCATGGCATCTTCTTCCCCTAGGTACAG |
| Os11g45740_Outer | GGCAGCTCTTCCCAGTCCTCTT |
| Os11g45740_Inner | GCCGGAGCTCAGCCGACGACAGCT |
